# Different Sequential Renal Involvement in a Patient With HIV and Hepatitis C: From HIV‐ or Leishmaniasis‐Related Collapsing Glomerulosclerosis to Direct‐Acting Antivirals’ Renal Amyloidosis

**DOI:** 10.1155/crdi/7937029

**Published:** 2026-01-28

**Authors:** María Adoración Martín Gómez, Mercedes Caba Molina, Elisa Fernández Fuertes, Inés Pérez-Camacho, Ana Belén Lozano Serrano, Rafael del Pozo Álvarez

**Affiliations:** ^1^ Nephrology Unit, Hospital Del Poniente, El Ejido, Almería, Spain; ^2^ Pathology Department, Parque Tecnológico de la Salud, Granada, Spain; ^3^ Internal Medicine Department (Infectious Diseases Unit), Hospital de Poniente, El Ejido, Almería, Spain; ^4^ Nephrology Department, Hospital Regional Universitario de Málaga, Málaga, Spain, hospitalregionaldemalaga.es

## Abstract

The paradigm of renal involvement in HIV patients has changed in recent years, from HIV‐associated nephropathy to nephroangiosclerosis, due to the increased survival of these patients and their comorbidities. Some of these are leishmaniasis and hepatitis C and their treatments, especially direct‐acting antivirals, which may induce reconstitution of the cellular immunity and activate a latent autoinflammatory disease. Case presentation: We present a 51‐year‐old Caucasian patient with chronic HCV liver disease and HIV Stage A3 who suffered from kidney disease throughout his life. In the first episode, he debuted with nephrotic proteinuria when he was not taking any treatment for HIV. Renal biopsy showed focal segmental glomerulosclerosis that could be due to HIV or other infectious‐related disease such as leishmaniasis. Whatever it is, the proteinuria responded to treatment for both infectious diseases. Nine years later, while the patient was on treatment with a new antiviral for HCV, he presented a complete nephrotic syndrome flare. A second biopsy showed amyloidosis A. The first biopsy was then reviewed, and minimal traces of amyloid were detected. Conclusions: Kidney involvement in HIV patients should be examined with high precision to detect any sign of different renal pathologies that may coexist. Comorbidities and their treatments might challenge and add to the differential diagnosis.

## 1. Introduction

Renal involvement in HIV patients can be multifactorial, and its paradigm has changed in recent years [1]. In the early​ stages of the HIV history, HIV‐associated nephropathy (HIVAN) was the most frequent renal manifestation of HIV, as the infection was barely controlled. Since the arrival of highly active antiretroviral therapy (HAART), its incidence has decreased, although it is still the nephropathy most frequently found in the biopsies of African patients [2].

Visceral leishmaniasis (VL) is endemic in the Mediterranean and Central African countries, with a seroprevalence ranging between 2% and 17%; it is a zoonotic infection with the dog being the main reservoir. In Spain, it is more frequent in HIV patients, especially among those with significant immunosuppression (CD4 lymphocytes below 200 cells/µL) [3]. Although there are large records describing renal involvement due to leishmaniasis in animals [4], in humans, the literature consists mainly of case reports. The finding most frequently described in both animals and humans is tubulointerstitial nephritis [5], closely followed by glomerulonephritis, in the form of mesangial, membranoproliferative, or membranous glomerulonephritis, collapsing focal segmental glomerulosclerosis (cFSGS), and cryoglobulinemia, among others [6–11].

On the other hand, thanks again to the use of HAART, the survival of HIV patients has increased, and HIV infection has turned into a chronic disease. Other acute and chronic conditions of different etiologies (infectious or their treatment, inflammatory, and cardiovascular) can then appear over time and eventually induce the development or reactivation of a new or latent secondary amyloidosis [12].

We present the case of a patient coinfected with HIV, HCV, and leishmaniasis who presented two different kidney pathologies during the course of his disease.

## 2. Case Presentation

We present the case of a 51‐year‐old Caucasian male. He presented to the emergency department in May 2009 with a 2‐month history of mechanical low back pain and asthenia without fever. He was a former intravenous drug user (IVDU) and suffered from chronic HCV liver disease with moderate fibrosis and HIV infection with advanced immunodeficiency although asymptomatic (CDC Stage A3 and WHO clinical Stage 1). He was not taking HAART and was lost to follow‐up in previous years. His evolution is depicted in​ Figure 1.

**Figure FIGURE 1:**
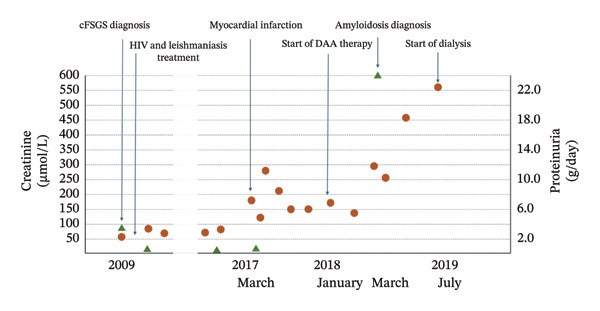
Timeline of events with creatinine and proteinuria levels.

On examination, he was afebrile, cachectic (body mass index of 15.57 kg/m^2^), and pale; his blood pressure was 117/76 mmHg, and his pulse was 83 bpm, with normal cardiorespiratory auscultation. An excavated abdomen and hepatomegaly were found, with no edema in the legs. Laboratory testing revealed moderate pancytopenia, low blood levels of iron, albumin, and total cholesterol and polyclonal hypergammaglobulinemia. Blood levels of electrolytes, glucose, coagulation tests, liver function, and triglycerides were normal. Renal test revealed normal creatinine (61.89 μmol/L) but a proteinuria of 3.47 g/day with inactive sediment. Tests for autoimmunity (ANA, anti‐DNA, anti‐ENA, ANCA, ASLO, RF, C3 and C4, and cryoglobulins) were negative, as well as the serologies for HBV, *Brucella, Leishmania*, CMV, and EBV. HIV viral load was 149,000 copies/mm3, HCV‐RNA viral load was 19,900,000 IU/mL, and CD4 lymphocyte count was 135 cells/mm3 (13%). An ultrasound examination showed enlargement of the liver and spleen, while the kidneys were normal and the transient elastography measured 7.4 kPa. A vertebral compression fracture was seen in the lumbar spine X‐ray. Renal biopsy was performed (Figure 2).

**Figure FIGURE 2:**
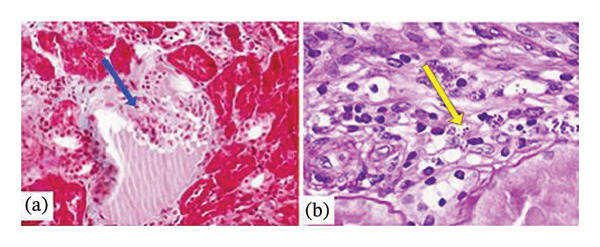
(a) Image corresponding to Masson’s trichrome stain showing the presence of wrinkled and collapsed glomerular capillary walls (arrow) with relative dilation of the urinary space (× 20 magnification). (b) In the interstitium, inflammatory foci are identified with macrophages, inside which leishmania (amastigotes) are observed (arrow) (PAS × 40).

Kidney biopsy 1 (17 glomeruli and 3 sclerosed). The nonsclerosed glomeruli showed swelling of the visceral epithelial cells, some degree of tangled collapse in others, and focal and segmental hyalinosis in another (Figure 2(a)). In the interstitium, occasional inflammatory foci were identified with macrophages, inside which amastigotes of *Leishmania* were observed (Figure 2(b)). Direct immunofluorescence showed weak (+1) segmental and diffuse positivity for C1q in glomeruli; intense (+3) global and diffuse positivity for C3 in mesangial areas, and to a lesser extent in capillaries; and mesangial positivity for IgG (0/+) and for IgM (+3). Immunohistochemical staining for SV40 virus and cytomegalovirus was negative. Following these data, the pathologic diagnosis was cFSGS associated with interstitial macrophage inflammatory foci with *Leishmania* amastigotes inside.

After these findings, *Leishmania* serological tests, polymerase chain reaction (PCR), and peripheral blood culture were performed, with the latter two testing positive for *L. infantum*. A diagnosis of glomerular and tubulointerstitial changes typical of cFSGS probably related to HIV nephropathy and leishmaniasis was made.

The patient received pharmacological treatment for leishmaniasis with parenteral amphotericin B as follows: 4 mg/kg daily for 25 days plus once weekly for 5 more weeks, receiving a cumulative dose of 120 mg/kg for 45 kg of weight (5400 mg). HIV treatment was resumed with tenofovir disoproxil fumarate and lamivudine.

He followed up in the infectious disease unit but did not keep his appointments with the nephrologist. His proteinuria improved to 0.3–0.6 g/day, and the urinary sediment showed just slight microscopic hematuria (5–10 RBC/HPF). In the following years, he continued on antiretrovirals but had to be treated for leishmaniasis again in 2010 and 2012. The relapses were related to the discontinuation of the leishmaniasis maintenance treatment due to irregular adherence to parenteral treatment and clinical follow‐up. After starting oral maintenance treatment with miltefosine for secondary prophylaxis, adherence improved and recurrences disappeared. Consequently, the patient’s immunovirological status improved, and miltefosine treatment was stopped in January 2013.

In the next years, he developed ischemic heart disease and chronic obstructive pulmonary disease (COPD) without bronchiectasis. Creatinine figures had remained stable until March 2017, when the patient suffered an acute kidney injury associated with an acute myocardial infarction, then recovered, and progressively deteriorated, with a mean creatinine of 138 μmol/L. Besides, he was successfully treated for hepatitis C with direct‐acting antivirals (DAA) from January to April 2018. In March 2018, renal function suddenly took an unexpected turn, with creatinine rising to 291.79 μmol/L and proteinuria to 24 g/day, developing a complete nephrotic syndrome. Autoimmunity markers were negative once more, and HIV infection was well controlled for the past recent years. A second kidney biopsy was performed (Figure 3) with 20 glomeruli (6 sclerosed). Deposits of an amorphous PAS‐negative substance was observed with global diffuse glomerular involvement and focal interstitial, peritubular, and also affecting arterioles’ walls (Figure 3(a)) but not with Jones’s Silver stain (Figure 3(b)). After staining with Congo red, it showed apple‐green birefringence (Figure 3(c)). Interstitial fibrosis and tubular atrophy were moderate. No amastigotes were found. Immunohistochemical staining was positive for amyloid A (Figure 3(d)). Direct immunofluorescence was negative. In light of these findings, the first renal biopsy was reviewed and very faint traces of amyloid A fibrils were detected (Figure 4).

**Figure FIGURE 3:**
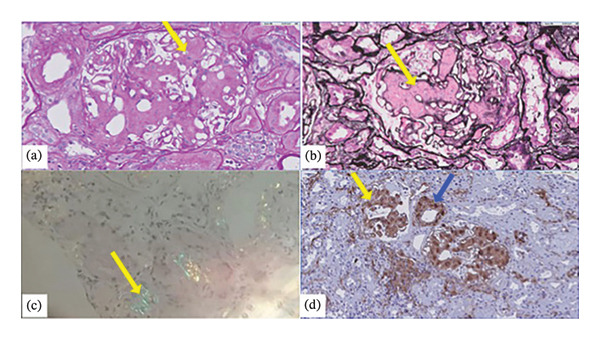
(a) Diffuse global glomerular deposit with weak PAS positivity (arrow) (× 40 magnification). (b) The deposit is not stained with Jones’s Silver stain (arrow) (× 40 magnification). (c) Amorphous deposits show positivity with Congo red staining and characteristic apple‐green birefringence when examined under polarized light (arrow) (20 × magnification). (d) After immunohistochemical study with staining for amyloid A, glomerular (yellow arrow), interstitial, peritubular, and arteriole walls (blue arrow) deposits were revealed, findings compatible with AA amyloidosis (magnification × 20).

**Figure FIGURE 4:**
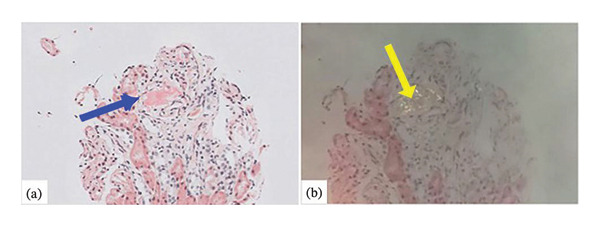
(a) Glomerular amyloid traces in the first biopsy (arrow) (Congo red, × 20). (b) Characteristic apple‐green birefringence when examined under polarized light (arrow) (× 20).

A comprehensive workout, including a positron emission tomography scan and a computed tomography scan (PET‐CT), was carried out to rule out secondary diseases, with no further findings. Despite his COPD, he did not suffer from recurrent respiratory infections, and no other subacute or chronic infectious process, unknown until that moment, was found through serological assessment. Although the PCR test for *Leishmania* remained negative, the patient received proper treatment for leishmaniasis once more since no active cause responsible for the secondary amyloidosis was found. Nonetheless, he evolved towards end‐stage renal disease (ESRD), entering dialysis in July 2019.

## 3. Discussion

This clinical case provides data from two kidney biopsies in the follow‐up of a patient with HIV and HCV coinfection, as well as other high‐quality diagnostic tests such as PET‐CT. Scintigraphy for amyloid P is not available in our region.

The name of collapsing glomerulonephritis was introduced in the 1970s within the classification of focal segmental glomerulosclerosis. Later, it was associated with HIV and other conditions, including leishmaniasis and hepatitis C [13, 14]. HIV patients commonly suffer from other coinfections, as in the case of our patient, either because they share the same transmission route (e.g., HCV) or because the immunosuppression due to HIV infections favors the acquisition of opportunistic diseases such as leishmaniasis.

In the patient in question, cFSGS could be secondary to HIVAN, leishmaniasis, or hepatitis C, since it has been described independently in all of them. Some data point toward HIVAN as the cause responsible for the cFSGS (absence of edema or nephrotic syndrome), while other findings point to leishmaniasis (a normal ultrasound examination, the amount of the mesangial IgM deposit, and a clinical presentation without renal failure). An electron microscope was not available to assess reticular inclusions, but treating HIV and leishmaniasis resolved the renal condition almost completely [15–19]. Hepatitis C would not be treated until 9 years later, so it could be assumed that it was not the cause of the glomerulopathy. More detailed information is displayed in Table 1.

**Table TABLE 1:** Differential diagnosis between HIV‐associated cFSGS, leishmania‐associated cFSGS, and renal AA amyloidosis.

	Clinical characteristics	Kidney ultrasound	Histopathology		
			Light microscope	Immunofluorescence	Electron microscope
HIV‐associated nephropathy	‐Nephrotic syndrome without edema or high blood pressure.	‐Increased renal echogenicity +++.	‐cFSGS.	IgM, C3 (+3), C1q (+1) in sclerosis areas.	‐No deposits.
	‐Renal failure (frequent).	‐Enlarged kidneys.	‐TI involvement.		‐Reticular inclusions (90%).
			‐Cystic dilatation.		
Leishmaniasis	‐Nephrotic syndrome.	‐Increased renal echogenicity ++.	‐cFSGS.	IgG, C3, IgM (+2) mesangial and membranous.	Subepithelial and mesangial electron‐dense deposits.
	‐ Renal failure (rare).	‐Normal‐sized kidneys.	‐TI involvement.		
Renal AA amyloidosis	‐Little to massive proteinuria. Nephrotic syndrome (rare).	‐Increased renal echogenicity ++.	‐Acellular Congo‐red positive deposits.	Serum amyloid A immunostain.	Fibrils randomly arranged, 8–12 nm in diameter.
	‐Renal failure (uncommon).	‐Enlarged kidneys.	‐TI involvement rarely.		

*Note:* TI: tubulointerstitial.

Abbreviation: cFSGS: collapsing focal segmental glomerulosclerosis.

HIVAN seems to be related to uncontrolled HIV infection, and that is the reason behind its decreasing incidence in the developed world after the appearance of HAART. On the other hand, regarding leishmaniasis, the relationship between the severity of the infection or the time of evolution and the renal involvement, as well as the pathogenesis of the nephropathy, is not clear. It seems to be mixed in terms of the host’s immune response and the ability of the parasite to resist macrophage action. Both cellular immunity, with macrophages and T cells expressing cytokines, and humoral immunity, with the polyclonal activation of B lymphocytes triggered by the parasite and the subsequent deposits of immune complexes, are involved [20].

The interaction between leishmaniasis and HIV is positive for both infections, facilitating the progression of each other through a synergistic effect against cellular immunity. Leishmaniasis in the HIV patient is characterized by some peculiarities: a less sensitive serological diagnosis, visualization of fewer immune complexes and a greater presence of the parasite in tissue samples, atypical clinical presentation and atypical locations, worse response to treatment, and a higher recurrence rate. Subclinical disease can occur equally in the different stages of HIV, irrespective of the number of CD4 lymphocytes. It may thus become a chronic disease with multiple recurrences that can lead to new renal flares [21, 22], even under successful antiretroviral treatment [23, 24]. For this reason, secondary chemoprophylaxis (chronic maintenance treatment for leishmaniasis) is recommended, particularly for patients with CD4 cell counts below 200 cells/mm3, although it may be still indicated over that limit. For this reason, our patient was empirically treated with antileishmanials—amphotericin B—once again during his second nephrotic episode.

In the literature, the cases of glomerulopathy described in coinfected patients resembling our case are varied and mostly attributed to leishmania due to presenting a low or undetectable HIV viral load but a deficient immune status with low CD4 counts, or because of presenting glomerular affectation nontypical of HIV [21, 22, 25–29]. The development of one type or another of glomerulonephritis, with or without immune complexes, may be influenced by the virological status of the patient, which determines a greater or lesser degree of immunosuppression.

With respect to the second biopsy, AA amyloidosis is caused by the abnormal transformation of amyloid protein A (SAA), considered an acute‐phase reactant, into amyloid fibrils. This transformation is activated by proinflammatory cytokines, secreted in the course of different conditions (autoimmune, infectious, neoplastic, etc.) that cause a chronic or prolonged inflammatory state over time. In the present case, several circumstances that could act as an amyloidogenic trigger have been described: HIV, HCV, leishmaniasis, and IVDU [30–32]. Apart from those, as we described previously, we did not find any other evident cause of the amyloidosis in our case.

There are reports of cases of amyloidosis secondary to HIV infection, especially in IVDUs with intercurrent skin infections and those not controlled virologically that suffer recurrent infections or coinfections, including leishmania, but also related to HIV infection itself, through the activation of molecules such as TNFa, IL 6, and NFk‐B [30–33]. Regarding the association of secondary renal amyloidosis and leishmaniasis, there are 2 cases described in 2006 and 2009 [31, 32], both occurring in patients with HIV infection. The rest of the cases are in experimental animals, without a clear pathogenic explanation. In our case, it is of interest that the virological status had been excellent for the last years and that he had abandoned the use of drugs long time ago as far as his physicians were aware of.

The mean time between the onset of the disease‐causing AA amyloidosis and the development of amyloidosis itself is around 2–7 years. In our case, HIV infection was not controlled for a few years, at least from 2005 to 2009. Since then, the patient was under HAART with undetectable viral loads. Leishmaniasis was diagnosed and treated in 2009, although we cannot rule out a silent infection before 2009, and he suffered from two overt clinical relapses, as discussed above. Finally, HVC infection was also diagnosed in 2009, although probably present from long before, and successfully treated in 2018.

In our case, despite the amount of amyloid material deposited in the kidney, the levels of serum amyloid A protein were within normal range (4 mg/L, normal < 5 mg/L). In the literature, the level of amyloid protein in serum is related to the prognosis of the disease. It is to be noted that the measurement of serum amyloid protein was made months after the histological diagnosis, when neither amyloidosis nor renal function were improving, but quite the opposite; therefore, an increased serum amyloid was to be expected. Experts in amyloidosis explain this phenomenon by the coexistence of other conditions damaging the kidney simultaneously or by an already established chronicity of the renal dysfunction when amyloidosis is diagnosed [34]. In our patient, 6 out of 20 glomeruli were sclerosed, and interstitial fibrosis and tubular atrophy were already moderate, so the latter is the most probable explanation.

Retrospectively, we asked ourselves how long our patient was affected by a latent AA amyloidosis. This entity may have set years ago before its abrupt debut, and some factor might had changed the course of the disease. This fact has been described physiopathogenically in relapses of renal amyloidosis caused by an inflammatory stimulus acting on residual deposits [34]. This stimulus is called “amyloid enhancing factor,” and the phenomenon has been verified experimentally [35, 36]. In our case, there were already traces of amyloid in the 2009 biopsy, but they did not become clinically evident until 9 years later. It could have been a very mild amyloidosis, related to HIV infection or to leishmaniasis, that remained latent after treating both conditions. But then, it suddenly becomes clinically apparent. However, it is to note that this late finding in a 2009 biopsy could reflect a confirmation bias.

It was not possible to find any cause allegedly responsible for the amyloid outbreak in this patient. The previous year, he suffered from ischemic heart failure, increasing his baseline serum creatinine levels, and in 2018, two months after initiating an antiviral treatment for hepatitis C, his kidney disease aggravated and his proteinuria was found to have skyrocketed. To date, there are no described cases of AA amyloidosis and DAA. Nonetheless, a reconstitution of the cellular immune system following this treatment has been described [37, 38]. In such reconstitution, the participation of monocytes, NK cells, CD4 and CD8 T lymphocytes may lead to an upregulation of IFN I/*α*, IL‐6 and to a downregulation of IFN type II/*γ* and III, IL‐10, among others, which may facilitate the reactivation of latent infections. Besides, IL‐6 has been postulated as a proinflammatory mediator in amyloidosis experimentally [36]. Other groups have tried interleukin 6 blockade with unconclusive results, presumably due to unknown and intervariable proinflammatory profile of their patients [39]. Hypothetically, DAA could have been the stimulus for our patient, thus facilitating a possible activation or reactivation of amyloidosis A. Should this hypothesis be true, treatment with tocilizumab might have been a useful option.

In reflecting on our findings, we must acknowledge the inevitable influence of retrospective bias, which can color our interpretation of past data. Particularly, there are some temporal uncertainties when it comes to amyloidosis sprout and kidney function decline as the patient at that time had lost follow‐up with the nephrology unit and urine tests were scarce. Consequently, our presentation of this case remains fundamentally speculative. By foregrounding these limitations, we invite readers to view the results as a steppingstone toward deeper investigation.

## 4. Conclusion

Renal involvement in HIV patients represents a wide range of diagnostic possibilities, including different infections that frequently coexist with HIV infection, as well as their own treatments. Renal biopsy stands out as an essential tool. The nephrotic syndrome in a patient with HIV, HCV, and leishmaniasis can typically be due to cFSGS, but secondary amyloidosis must also be considered. Regarding amyloidosis, we hypothesize it might be explained in conjunction with silent chronic conditions such as leishmaniasis. The role played by reactivation of immunity after the treatment of HVC with the new antivirals is yet unclear.

## Author Contributions

María Adoración Martín Gómez has written the first draft and coordinated the rest of the authors. Mercedes Caba Molina, Elisa Fernández Fuertes, Inés Pérez‐Camacho, and Ana Belén Lozano Serrano have contributed with ideas and literature review, as well as the final revision of the manuscript. Rafael del Pozo Álvarez has revised the original draft, all versions of the manuscript, figures, tables, and other necessary changes for journal adaptation.

## Funding

No funding was received for this manuscript.

## Ethics Statement

This study was carried out in accordance with the principles and ethical standards of the Declaration of Helsinki (revised version of Fortaleza, 2013) and was approved by the Ethics and Research Committee of the Study Coordinating Center of Almería, Code 145/2021.

## Consent

No written consent has been obtained from the patient. This paper was written after the patient’s death and there are no living relatives to ask for consent. Nevertheless, the patient told his infectologists and coauthors of this article that he would be proud that his case could be used for clinical research or published for educational purposes, for it was his will to collaborate with science.

## Conflicts of Interest

The authors declare no conflicts of interest.

## Data Availability

Data sharing is not applicable to this article as no datasets were generated or analyzed during the current study.
